# Papain gel containing methylene blue for simultaneous caries removal and antimicrobial photoinactivation against *Streptococcus mutans* biofilms

**DOI:** 10.1038/srep33270

**Published:** 2016-09-19

**Authors:** Zenildo Santos Silva Jr., Ying-Ying Huang, Lucas Freitas de Freitas, Cristiane Miranda França, Sergio Brossi Botta, Patrícia Aparecida Ana, Raquel Agnelli Mesquita-Ferrari, Kristianne Porta Santos Fernandes, Alessandro Deana, Cintia Raquel Lima Leal, Renato Araujo Prates, Michael R. Hamblin, Sandra Kalil Bussadori

**Affiliations:** 1 Massachusetts General Hospital, Wellman Center for Photomedicine, Boston, MA 02114, USA; 2 Harvard Medical School, Department of Dermatology, Boston, MA 02114, USA; 3 Postgraduate Program in Biophotonics Applied to Health Sciences, Nove de Julho University (UNINOVE), São Paulo, SP, Brazil; 4 Postgraduate Program in Bioengineering of São Paulo University, São Carlos, SP, Brazil; 5 Center of Engineering, Modeling and Applied Social Sciences, Federal University of ABC, Sao Bernardo do Campo, SP, Brazil; 6 Harvard-MIT Division of Health Sciences and Technology, Cambridge, MA 02139, USA

## Abstract

This study intended to evaluate the effects of a papain-gel with a red-light absorbing pigment (methylene blue – MB) to mediate photodynamic therapy (PDT) against *Streptococcus mutans* biofilms. The PapaMBlue was compared with free MB to generate reactive oxygen species using fluorescence probes (SOSG and HPF). PDT (660-nm light) was carried out against *S. mutans* biofilms grown on either plastic dishes or on collagen membrane and assayed by CFU, live-dead staining using confocal microscopy, transmission electron microscopy and H&E staining for collagen films. Cytotoxicity and subcellular localization was studied in human fibroblasts. Sponges of bioabsorbable type I collagen membrane were exposed to papain based gel, irradiated with laser and analyzed about their integrity by ATR-FTIR. The PapaMBlue produced higher amounts of singlet oxygen and hydroxyl radicals than free MB, possibly due to better disaggregation of the dye in solution. The PapaMBlue antimicrobial effects on biofilms proved to be capable of reducing the *S. mutans*. Both MTT and PrestoBlue assays showed higher cell viability and metabolism scores in fibroblasts treated with PapaMBlue and MB, possibly due to stimulation of mitochondrial activity and that collagen triple helix is unaffected. The PapaMBlue is equally effective as MB in destroying *S. mutans* biofilms growing on plastic or collagen without affecting fibroblasts.

Dental caries (tooth decay) is the most prevalent dental disease in humans worldwide^1^. The development of carious lesions stems from a dynamic process mediated by acid produced by cariogenic bacteria (the most prevalent are *Streptococcus mutans, Streptococcus sobrinus* and in deep carious lesions, *Lactobacilli*), eventually resulting in de-mineralization and damage to the tooth structure^1^,^2^.

Recent advances in restorative dentistry have emphasized the development of materials and minimally invasive procedures for debridement of decay and preservation of remaining sound dental tissue before restoration. Chemo-mechanical combination methods of caries removal have been increasingly investigated as an option for minimally invasive treatment^3^.

In order to maintain pulp vitality, carious lesions are partially removed, thereby allowing the preservation of sound tooth structure with the maximum amount of tissue capable of re-mineralization^2^,^3^. This method is currently considered the treatment of choice for deep carious lesions provided that certain treatment principles are observed^4^.

Chemo-mechanical agents for caries removal, rely on the action of proteolytic enzymes, such as papain, to further degrade the partially demineralized and altered dentin matrix that has been previously exposed to bacterial action (infected dentin), thus facilitating its removal and preventing damage to underlying tissues (affected but non-infected dentin)^5^. Papacárie is a gel composed of papain and chloramine^3^,^6^,^7^, the latter component has disinfecting properties^8^, while papain is a plant-derived cysteine protease with broad proteolytic activity, with similarities to human pepsin. The combination acts as a bactericidal, bacteriostatic and presents anti-inflammatory properties acting as a debriding agent that does not damage sound tissue^9^,^10^. An *in vitro* study using the technique of attenuated total reflectance Fourier-transform infrared spectroscopy (ATR-FTIR) demonstrated that papain-gel did not lead to the degradation of collagen and this product could be safely used in minimally invasive dentistry. As the integrity of sound collagen is preserved after the application of the papain-based gel, this product is indicated for the selective removal of infected dentin, leaving the underlying affected dentin intact and capable of re-mineralization^11^. Papacárie gel underwent initial studies over the last ten years^12^ in a children’s dental clinic. Due to promising laboratory and clinical results^2^,^3^,^6^,^7^,^9^,^10^, the clinical trials were expanded into other applications such as removal of decay (cavities) in patients with special needs^13^, and in adults^14^.

A controversy point of this gel is whether it reduces the bacteria colonies present in the dental tubules of affected dentin. Those bacteria products are harmful to the pulp, mainly in deep cavities, where the remaining thin layer of dentin is less rigid, and if removed excessively there is a chance of a pulpal exposition. When bacteria and their products (such as acid and released LPS in the dentinal fluid) come in contact with the pulp they generate an inflammatory process affecting fibroblasts and odontoblasts. This inflammation (even if subclinical) leads to decreased reparative properties of the dental pulp and predisposes to future degeneration, fibrosis and/or pulpal abscesses. Currently, one of the main goals of the researchers in dentistry is to preserve the potentially remineralizable affected dentin to increase the mechanical resistance of the remaining tooth, avoiding fractures and failures in the restoration.

A change in Papacárie composition was suggested in order to improve its properties and applicability, so it might be used as an agent for removal the necrotic dentin tissue and also as an antimicrobial agent. This change was performed by adding a photosensitizer that could be activated by light in photodynamic therapy (PDT) and the interaction between the gel and the light resource, regarding that hypothesis to potentiate the antibacterial effect of Papain gel added the methylene blue (Fig. 1).

Antimicrobial photodynamic therapy (aPDT) is a promising treatment to inactivate microorganisms. It employs a non toxic dye that can be activated by light, a light source of a specific wavelength and an environment in the presence of oxygen. When the photosensitizer is light-activated it induces the formation of reactive oxygen species (ROS). Two parallel pathways generate these ROS: type I reaction, which involves electron transfer to oxygen primarily forming superoxide and finally hydroxyl radicals, and type 2 reactions, which comprises energy transfer to ground-state triplet oxygen and production of singlet oxygen^15^. Recent studies demonstrated that aPDT is an encouraging adjunctive therapy to the treatment of caries lesions, mainly in the minimally invasive approach to preserve dental tissue and favor its repair^16^,^17^.

Methylene blue (MB) is well known to act by the type 1 mechanism (generating ROS including hydroxyl radicals) and also by type 2 mechanism (producing singlet oxygen). It has received regulatory approval and has been widely used in dentistry as an antimicrobial photosensitizer in periodontitis^15^, peri-implantitis^16^ and endodontic treatment^17^. It was proposed that PDT would not cause damage to pulpal tissue, and could be applied safely in deep carious lesions^15^,^16^.

The aim of this study was to evaluate cytotoxicity, phototoxicity, generation of reactive oxygen species and collagen type I degradation with microstructure analysis by this new photoactivated gel composition and caries lesion inducted by bacteria biofilm.

## Material and Methods

**Figure 2:**
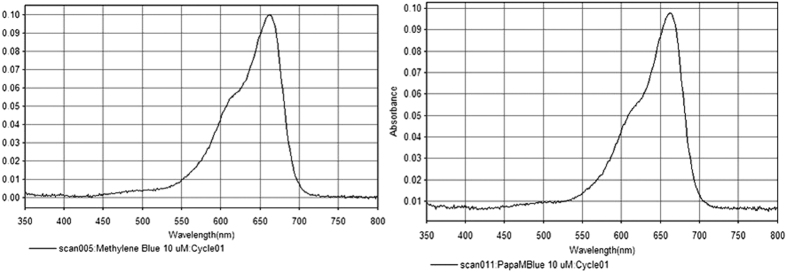
Absorption spectra of (**A**) MB and (**B**) PapaMBlue recorded at 10 μM in water showing alike patterns.

PapaMBlue and methylene blue stock solutions were made in dH2O at 1 mM concentration^18^,^19^. Solutions were stored for a maximum of 2 weeks at 4 °C in the dark. The absorption spectra were recorded at 10 μM in H_2_O using an Evolution 300 UV-Vis spectrophotometer (Thermo Scientific). The light source used was a broad-band lamp fitted with a band-pass filter (LumaCare Newport Beach, CA, 660+/−15 nm at an irradiance of 149 mW/cm^2^). It was found that both MB and PapaMBlue present similar absorption spectra (Fig. 2).

### Reactive Oxygen Species Assays (HPF and SOSG)

A solution of MB (1 μM, 50 μL) or PapaMBlue (1 μM MB equivalent, 50 μL) in PBS was added to 50 μL of a 20 μM solution of 3′-p (hydroxyphenyl)-fluorescein (HPF, Molecular Probes) in PBS in 96-well plates. Red light (LumaCare, 660-nm, 60 mW/cm^2^ output power density, doses 2 J/cm^2^, 5 J/cm^2 ^and 10 J/cm^2^) was delivered with fluorescence measurement (excitation wavelength of 490-nm and emission at 515-nm) taking place after each light dose (Spectramax M5, Molecular Devices). The fluorescence detected corresponds to the probe that has reacted with the hydroxyl radicals produced by the photoexcited sample, giving a fluorescent product.

**Figure 3:**
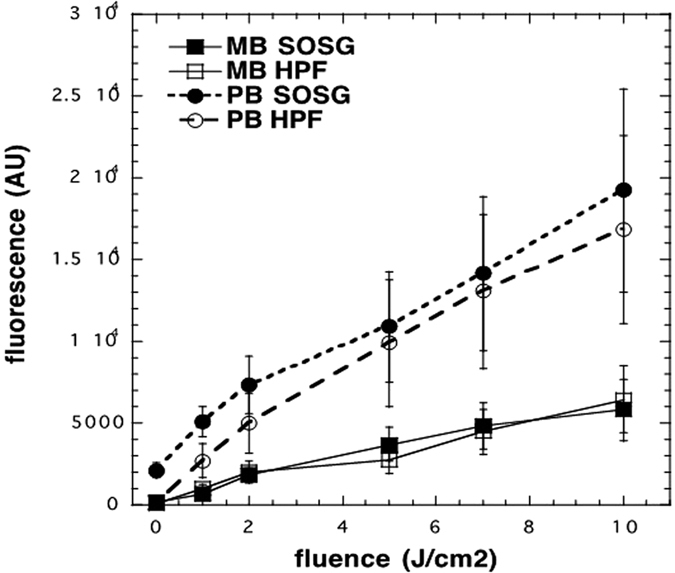
(*ROS*) probes (*HPF* and *SOSG*). MB or PapaMBlue were present at 1 μM MB equivalent. Probes were present at 20 μM. Mean plot of each group (±standard error) for different radiant energy exposures.

Singlet Oxygen Sensor Green (SOSG) probe (Molecular Probes) was used in a similar manner to that described for HPF. MB or PapaMBlue were used at 1 μM with SOSG at 20 μM. The same light irradiation parameters were used with fluorescence measurement at an excitation wavelength of 504-nm and emission at 525-nm. The fluorescence detected corresponds to the probe that has reacted with the singlet oxygen produced by the photoexcited sample, becoming a fluorescent product (Fig. 3).

### Cytotoxicity studies with human dermal fibroblasts

**Figure 4:**
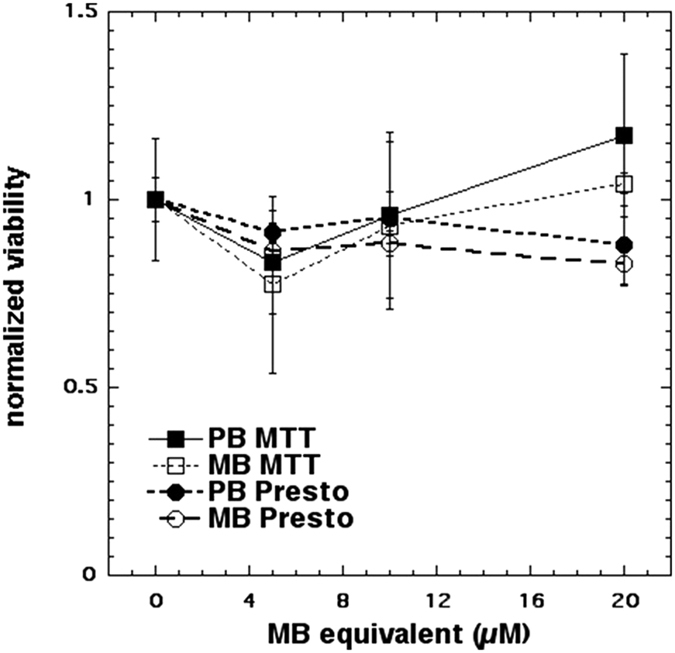
Viability of fibroblasts by MTT and PrestoBlue. MTT assay with MB and PapaMBlue at different concentrations (5–20 μM), normalized to control group without any treatment. PrestoBlue assay with MB and PapaMBlue at different concentrations (5–20 μM), normalized to control group without any treatment. Means ± standard error.

Normal human dermal fibroblasts (ATCC^®^ PCS201012™) were cultured in DMEM medium in 96-well plates, 5000 cells in each well (dark walls and glass bottom coated with 10% poly-D-lysine) and were left in the incubator for 24 h, at 37 °C and with 0.5% CO_2_ atmosphere. After 24 h the cell were incubated with culture medium containing 20 μM of MB or PapaMBlue for 4 hours. MTT assays were carried out according to the manufacturer’s instructions using 10 μL of 5 mg/mL solution in PBS per well. After 4 h the medium was removed and 100 μL of DMSO was added, then the plates were read in the microplate reader (Spectramax M5, Molecular Devices) absorbance at 570-nm. For the PrestoBlue assay, the cells were incubated for 4 h, with 10 μL of 10% dilution of the reagent added to 100 μL of DMEM medium. The reducing environment within viable cells converts the PrestoBlue reagent to an intensely red-fluorescent dye (excitation 560-nm and emission 590-nm). There was no difference in the cell viability among the groups (Fig. 4).

### Confocal Fluorescence Microscopy for Subcellular Localization

**Figure 5:**
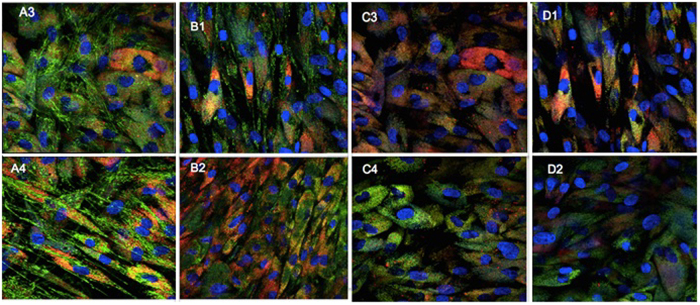
Confocal microscopy of fibroblasts with mitochondrial and lysosomal-specific probe. To stain organelles and localization of mithocondia in live cells used MitoTracker Green, Methylene Blue or PapaMBlue, and Hoesch. A3-PapaMBlue 10 μM Dark, A4-PapaMBlue 10 μM PDT. B1-methylene Blue 10 μM Dark, B2-methylene Blue 10 μM PDT. To localization lysosome LysoTracher Red, internalization of P.S methylene blue or PapaMBlue. C3 PapaMBlue 10 μM Dark, C4-PapaMBlue 10 μM PDT. D1-methylene blue 10 μM Dark, D2- methylene blue 10 μM PDT.

Human dermal fibroblasts (ATCC PCS-201-012, 5,000 cells) were cultured for 24 h on 1.5 high performance cover glasses (0.170+/−0.005 mm) coated with poly-D-lysine. After 24 h PapaMBlue or MB with a concentration 10 μM was added for 20 minutes drug light interval. After that, the cells were washed 3× with PBS and the medium was replaced (DMEM) and incubated for 1 h. After incubation the light groups were irradiated using the LumaCare lamp, 660-nm, 97 mW/cm^2^ output power 20 J/cm^2^, 15 min). Fluorescent probes were used to stain the cellular organelles were MitoTracker Green to stain mitochondria (excitation 490-nm and emission 516-nm, 50 nM), and LysoTracker Red to stain lysosomes (Fig. 5), (excitation 577-nm and emission 590-nm, 75 nM) and Hoechst trihydrochloride trihydrate to stain nuclei, (excitation 350-nm, emission 510-nm, 4 ug/ml, 40 μL). All probes were incubated for 45 minutes, the medium was removed 100 μL of Live Cell Imaging Solution (Molecular Probes), was added to provide better clarity, signal-to-noise ratio, and cell viability.

### PDT on *Streptococcus mutans* biofilms grown on plastic

The bacterial strains of *Streptococcus mutans* (ATCC 25175) were grown on incubator in agar plate (with BHI supplemented with 1% sucrose, after that step 24 h, 3 colonies of were removed and added to 8 mL BHI with 1% sucrose and the liquid culture was grown overnight 18 h in a shaker incubator (New Brunswick Scientific, Edison, NJ) at 120 rpm under aerobic conditions at 37 °C were incubated statically at 37 °C in air with 5% CO_2._ For the well preparation biofilm was used sterile 24-well plates, (SIGMA, ultra-low attachment, polystyrene, flat bottom, sterile). After 18 h the culture was centrifuged (15 minutes at 3500-rpm), the supernatant was discarded, the pellet was resuspended in 5 mL of sterile PBS. The samples were adjusted to an optical density of 0.8 at 600-nm (10^6 ^CFU/mL) (Spectromax). For biofilm formation, 100 μL inoculum of bacterial suspension, was added to individual sterile Petri dishes filled with 1 mL of BHI broth supplemented with 1% sucrose for 48 h at 37 °C, not shaking. After 48 h the cells were washed three times and fresh 1 mL of BHI broth supplemented with 1% sucrose added until biofilm maturation was complete at 72 hours^20^.

**Figure 6:**
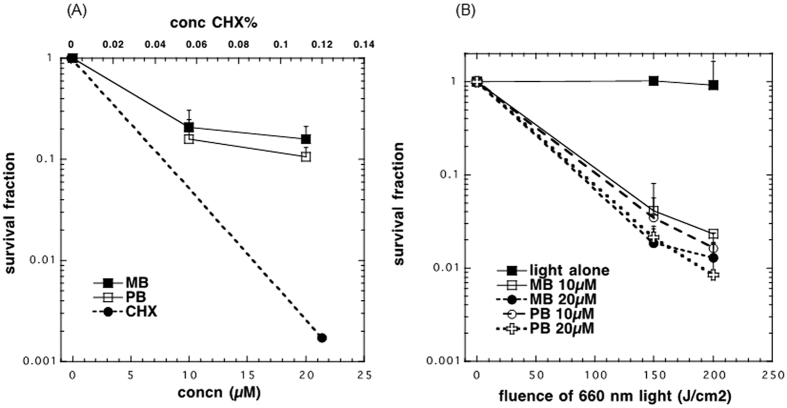
CFU reduction of biofims on plastic. (**A**) Survival fractions compared to absolute control of dark groups. Chlorhexidine 0.12% (CHX), MB 20 μM and PapaMBlue 20 μM (PB) in dark. (**B**) Survival fractions compared to absolute control of groups receiving light (0, 150 and 200 J/cm^2^). Light alone, MB 10 and 20 μM, PB 10 and 20 μM. Means ± standard error.

The groups tested are shown in Table 1 (G1, G3, G4, G5, G6 and G7) was dark control, no light treatment, the PDT groups (G2, G8, G9, G10 and G11), chlorhexidine group 0, 12% (G3), a gold standard against antiplaque agents are measured with properties and limitations of the molecule can ensure that the efficacy of the agent is maximized and the side effects are minimized allowing it to rightly remain the gold standard. (Table 1 and Fig. 6). Photosensitizers were added 10–20 μM. The drug light interval (DLI) was 30 minutes. PDT used light delivery of 150 J/cm^2^ during 53 min. and 200 J/cm^2^ during 70 min). To remove and disaggregate the bacteria from the surface of the well-plate was sonicated for 60 s (Bransonic 2510 Ultrasonic Cleaner, AC input 115 V, 2.8 L), to remove more than 99.9% of the biofilm bacteria with the sonication process. Serial dilution were carried out in 90 μL PBS (96 well plates) with 10 uL of inoculum, down to 10^−6 ^log. After the serial dilution 10 μL of inoculum of each dilution was added to BHI agar plates with 1% sucrose and were incubated at 37 °C for 24 h with 5% CO2, (Fig. 6).

### Fluorescence confocal microscopy of PDT of biofilms grown on glass

Following the same procedure the *S. mutans* (ATCC 25175) biofilms were grown in glass-bottom dishes (4-Chamber Glass Bottom Dish, 35 mm diameter) for 48 h at 37 °C. After 48 h, the cells were washed three times and replaced with 1 mL of BHI broth supplemented with 1% sucrose until biofilm maturation was complete after 72 hours. After 72 h the cell density, determined by CFU counting, was 2 × 10^7 ^cells/cm^2^. The 72 h biofilm was visualized using the LIVE/DEAD confocal fluorescent microscopy showing a single layer of cells with the clusters covering approximately 30% of the glass surface. The well dishes were incubated with (PapaMBlue) 20 μM for 30 minutes (DLI) and the light delivered (LumaCare 660 nm, 149 mW/cm^2^, 200 J/cm^2^ during 70 min).

**Figure 7:**
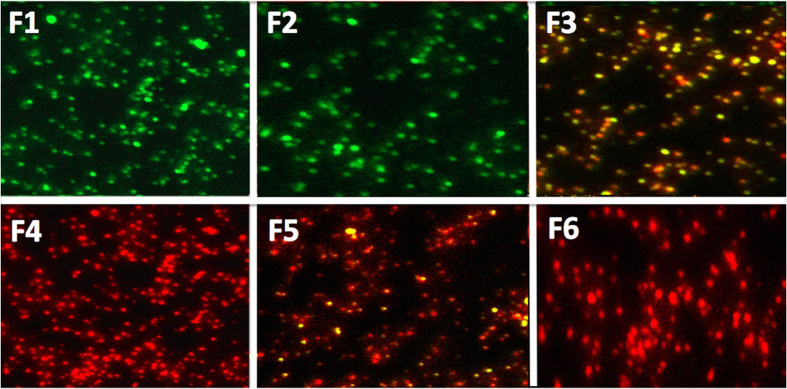
Confocal microscopy of biofilms on glass with live-dead stain. F1, No treatment; F2, light alone; F3, PapaMBlue 20 μM; F4, Chlorhexidine 0.12%; F5, PapaMBlue 20 μM 150 J/cm^2^; F6, PapaMBlue 20 μM 200 J/cm^2^.

Live/Dead BacLight (Bacterial Viability Kit) was then added according to the manufacturer’s instructions. The Syto9 stain was used with 480-nm excitation and 500-nm emission, to stain living bacterial cells green. The propidium iodide stain (490-nm excitation and 635-nm emission) was used to stain dead bacterial cells red (Fig. 7).

### PDT on *Streptococcus mutans* biofilms grown on collagen films

**Figure 8:**
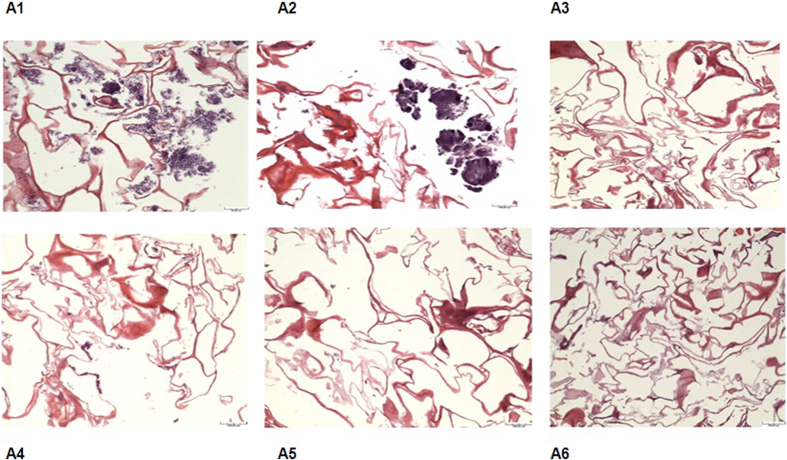
(*H&E*) staining of biofilms on collagen. (A1) Dark control, (A2) Light alone, (A3) PDT with MB 20 μM, 150 J/cm^2^, (A4) Chlorhexidine 0.12%, (A5) PDT with PapaMBlue 10 μM, 150 J/cm^2^, (A6) PDT with PapaMBlue 20 μM 150 J/cm^2^.

Sponges in the form of bioabsorbable membranes made of type-I collagen from bovine Achilles deep tendons (Technodry Liofilizados Médicos, Brazil) were cut with a sterile biopsy punch to obtain discs measuring 5 mm in diameter and 2 mm in thickness. The membranes were sterilized, and added to 1 mL of BHI broth supplemented with 1% sucrose and 100 μL inoculum of a suspension of *S. mutans* (ATCC 25175), (10^6 ^CFU/mL) at 600-nm, (Spectromax), following the previously protocol, were incubated in individual sterile Petri dishes for 48 h at 37 °C. After 48 h, the films were washed three times and replaced with 1 mL of (*BHI*) broth supplemented with 1% sucrose until biofilm maturation was complete in 72 hours. In different groups (n = 4 per group). Photosensitizers were added (PapaMBlue, 10 and 20 μM), and pure MB (20 μM), with a drug light interval of 30 min chlorhexidine group 0,12%, as the gold standard against antiplaque agent can ensure that the efficacy of the agent is maximized and the side effects are minimized allowing it to rightly remain the gold standard to control positive. The groups (A1, A2,) were dark control, group A2 light alone control, PDT groups (A3, A4, A5 and A6). The light parameters were applied (LumaCare, 660-nm, 149 mW/cm^2^, 150 J/cm^2^ during 53 min (Fig. 8).

### Transmission electron microscopy (TEM)

**Figure 9:**
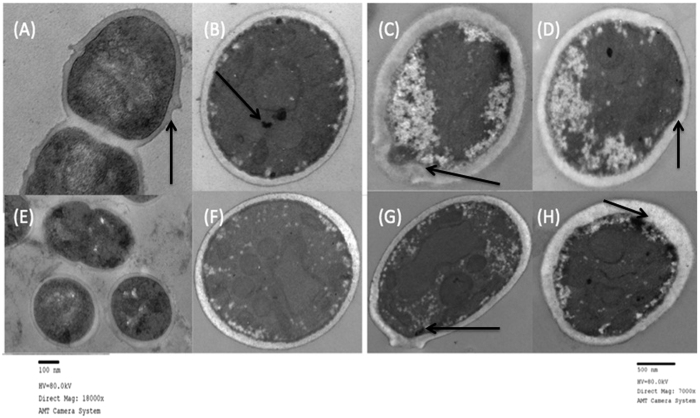
(*TEM*) of biofims on collagen. (**A**,**E**); Dark control. Cell envelope and membrane have normal morphology. (**B**,**F**); Light only. Intracellular granulation can be seen. (**C**,**D**) PDT with PapaMBlue 10–20 μM, 200 J/cm^2^. Membrane ruffling and degradation can be seen. (**G**,**H**); PDT with methylene Blue 10 μM, 200 J/cm^2^, membrane ruffling and degradation can be seen. Scale bars: (**A–D**, **F–H**) = 500-nm. (**E**) = 100 nm.

The study with the collagen membrane grown *S. mutans* using transmission electron microscope (TEM) analysis was applied to see how the microorganisms could be shown after PDT with PapaMBlue. Consequently, there have to be evidence of nanophase materials using collagen membrane, the atomic-resolution real-space imaging of nanoparticles. The experimental tissue preparation for (*TEM*) procedure, the samples was fix as small tissue of collagen type I. The morphology of bacteria analysis was carried out in the analysis, with (*TEM*). The position with respect to cellular ultrastructure, as well as the presence and morphology membrane could be observed between the groups, (Fig. 9). Such application in structural determination of shape-controlled nanocrystals and their assemblies. By forming a nanometer size electron probe, (*TEM*) is unique in identifying and quantifying the chemical and electronic structure of individual nanocrystals. *In situ* we could visualize with (*TEM*) how characterizing and measuring the thermodynamic membrane of bacteria beside the mechanical properties of individual nanostructures. For this specimen was former into smaller pieces (~1 mm^3^), and fixing in excessive fresh K2 fixative (5–10 times tissue volume) at 4 °C overnight, the samples were bring in fixative to Photopath within 24 h of fixing, as planktonic cells were grown in membrane of collagen type I, into to plate/dish, after the treatment or control group, the cells were in K2 fixative for 10 min at RT, then gently scrapped from the membrane. (*TEM*) morphology of the bacteria was carried out in the Wellman Center for Photomedicine, the images were recorded at 80 kV with a Jeol 1011 electron microscope. Scale bars: 500 nm. The position with respect to cellular ultrastructure, as well as the presence and morphology membrane could be observed between the groups, (Fig. 9).

### Caries lesion inducted by bacteria biofilm

**Figure 10:**
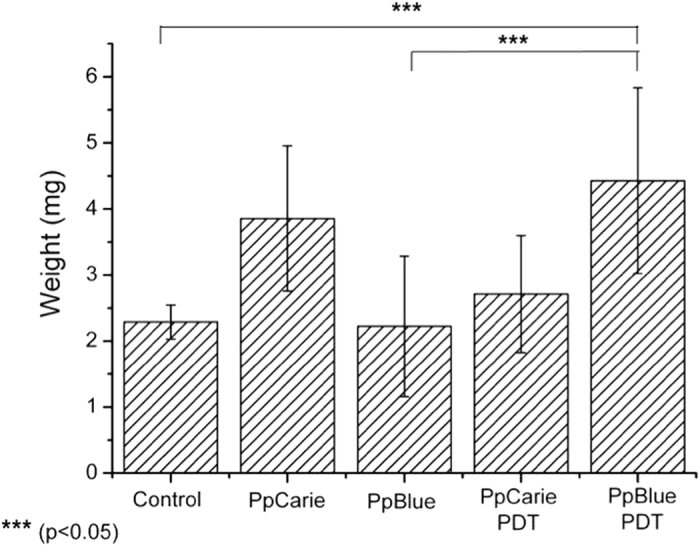
PapaMBlue combined to irradiation demonstrated to be more effective in the removal of the caried tissue in comparison with the control group and the PapaBlue group without irradiation.

Microbial biofilm were formed on bovine dentine slabs for 96 h and the current biofilm model was adapted from Fernández *et al*.^21^. Samples, 7 × 4 × 1 mm in size, were obtained from the crown and cervical roots of bovine incisors^21^. Since all samples were obtained post mortem from disposable parts of animals grown for commercial slaughter purposes at Frigobet, this work don’t require approval from the animal ethics committee (The Brazilian law number 11.797 that regulates animal procedures)^22^. Following the cut of the samples, they were placed on distillated water and then it was sterilized in autoclave. *S. mutans* ATCC 25175 grown on microaerophilic environment for 48 h at 37 °C onto culture medium brain-hart infusion (BHI). The slabs were immersed for 30 min in filtered human saliva to allow acquired pellicle and enable microbial adhesion. Tooth samples were incubated with *S. mutans* for 8 h on BHI broth containing 1 mM glucose. Over the next three days, biofilms were exposed to 10% sucrose solution for 3 min. 8 times a day. Following caries lesion formation, the samples were submitted to the treatments with PBS (n = 5), Papacarie (n = 5), PapaMBlue (n = 5), and more two groups with LED irradiation, named as Papacarie + PDT (n = 5) and PapaMBlue + PDT(n = 5). The sample were treated by eat condition for 30 s of contact and Papacarie+PDT and PapaMBlue + PDT were irradiated with 20 J/cm^2^, for 120 s by red LED (λ = 660 ± 15 nm) with irradiance adjusted to 0.167 W/cm^2^. Thereafter, samples were washed on PBS and biofilm was detaching from slabs on a vortex. The slabs were immersed on 2% chlorhexidine for 5 min affected dentin was removed using a dental curette. The removed dentin was weighted and data were analyzed (Fig. 10).

### Analysis of the effects of Papacárie and PapaMBlue on collagen microstructure

For this analysis, it was used 25 discs of sponges of bioabsorbable membranes. The samples had 5 mm in diameter and 2 mm in thickness, and were composed by type I collagen from bovine Achilles deep tendon. After preparation of the disks, they were randomly distributed among five experimental groups (n = 5), according to the Table 2.

In G1 group (control group), each sample was washed with Milli-Q water (Millipore, Bedford, MA, USA) for 30 seconds and, after that, dried with absorbing paper (Whatman no.6, Whatman International, England). In G2 group, each membrane was fully covered with the papain-based gel, for 5 minutes; after this time samples were abundantly washed with Milli-Q water for 30 seconds and dried with absorbing paper. In G3 group, samples were fully covered with the papain-based gel PapaMBlue for 5 minutes and then irradiated for 8 minutes using a red laser light that emits at wavelength of 654–662 nm, irradiated with 20 J/cm^2^. Subsequently, samples were abundantly washed with Milli-Q water for 30 seconds and dried with absorbing paper.

All treatments were performed immediately before the analysis of each sample by infrared spectroscopy in order to avoid dehydration or excessive water incorporation in samples, which can alter the spectra.

**Figure 11:**
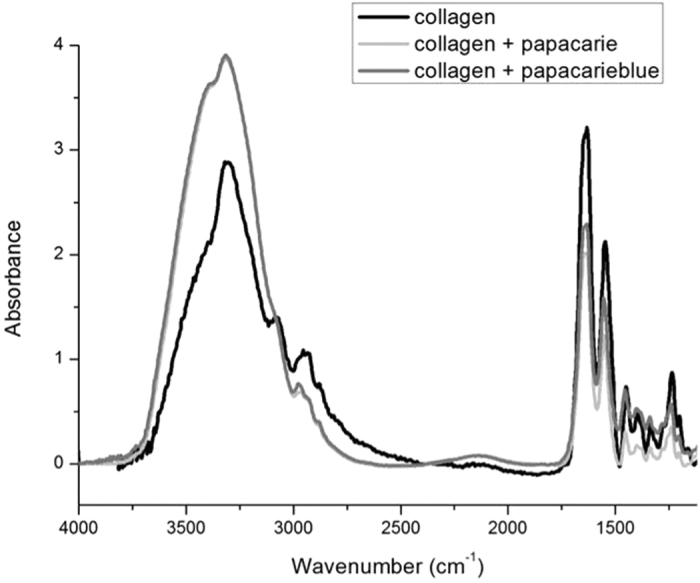
FTIR. Average of infrared spectra of pure type-I collagen membrane and after treatment with Papacárie and PapaMBlue in the region of 1000–4000 cm^−1^.

The compositional analysis of all samples was performed using the Attenuated Total Reflectance technique of Fourier Transformed Infrared Spectroscopy (ATR-FTIR, Varian 610, Agilent Technologies, CA, USA), using a diamond crystal coupled to the spectrometer, as previously described by Júnior *et al*.^11^. The ATR-FTIR spectra of each sample were obtained at a resolution of 4.0 cm^−1^, with 80 scans in the range of 4000 to 500 cm^−1^. A single spectrum was collected for each sample after the subtraction of the background. After data acquisition, the spectra had vetorial normalization after the subtraction of a baseline and the peak heights of each infrared band were analyzed. The recording and conversion of absorption spectra were performed using the Varian Resolutions Pro software program and the subtraction of the baseline, the normalization of the spectra and the analysis of the peak heights were done using the Origin Pro 8 software program (Fig. 11).

### Statistical analysis

The ratio of the MTT and PrestoBlue test data was calculated with respect to the dark group (% of control). Since data was not normal, the Kruskal-Wallis test was used and there was no statistical difference among the MTT analysis of the groups (p = 0.8 and 0.4, respectively).

The survival fraction of the number of colony forming unit in biofilm of *S. mutans* after PDT was analyzed using one-way analysis of variance (p < 0.0001), and Tukey’s Multiple Comparison Test. All groups presented statistical significance (p < 0.05).

For the analysis of the integrity of collagen triple helix, it was considered the peak absorbance ratios of 1235 cm^−1^/1450 cm^−1^ peaks, in which a ratio closer to 1 denotes the maintenance of the integrity of amide III and CAH bond of the pyrrolidine ring of the type I collagen triple helix^23^.

## Discussion

The present study is an important first step to launch a new product proving that the addition of methylene blue to Papacarie is reliable because: (1) it it does not change the absorption spectra of Papacárie (Fig. 2); (2) it preserves the biocompatibility of the gel (Fig. 4); (3) the PapaMBlue can be internalized by the cells and be identified in the mitochondria and lysosomes (Fig. 5); (4) the gel with MB is able to produce *ROS* (Fig. 3); (5) PapaMBlue is capable of reducing the *S. mutans* CFU of biofilm on plastic and (6) also in caries lesion inducted by bacteria biofilm (Fig. 10) and (7) does not degrade collagen (Figs 8, 9 and 11).

Methylene blue (MB) is a promising photosensitizer dye due to its high quantum yield (ΦΔ≈0.5) and long absorption wavelength (λ max = 664 nm), enabling a better light penetration depth in live tissue, as well as due to its high solubility in aqueous media. MB has by far been the most studied antimicrobial photosensitizer both “*in vitro”* in animal studies of infection and in human clinical studies particularly in dentistry. Figure 2 shows the absorption spectra of MB and PapaMBlue that are essentially identical. The key issue that was investigated in this study was to answer the question of whether a combination of MB with the Papacárie - papain gel would perform equally well (or even better) than free MB as an antimicrobial photosensitizer to destroy *S. mutans* biofilms.

The first step was to evaluate whether the gel possessed the ability to produce reactive oxygen species (*ROS*) upon illumination. ROS refers to a group of molecules such as superoxide anion, hydroxyl radicals, hydrogen peroxide and singlet oxygen^24^. ROS play key roles in many pathogenic processes, including carcinogenesis, inflammation and ischemia-reperfusion injury^25^, and signal transduction^26^,^27^,^28^,^29^. Several methods, including electron spin resonance^30^ and chemiluminescence^31^, have been developed to detect (*ROS*), but fluorescence detection is superior in terms of high sensitivity and experimental convenience. To detect singlet oxygen (^−1^O_2_), the commercially available fluorescent sensor named Singlet Oxygen Sensor Green (SOSG) has been widely used in many studies applying photodynamic therapy, as dyad composed of fluorescein and an anthracene moiety^32^. To detect hydroxyl radical we used 4-hydroxyphenyl-fluorescein that releases free fluorescein upon attack of the phenyl ring by hydroxyl radicals^33^.

Figure 3 shows that for the formation of both singlet oxygen (SOSG) and hydroxyl radical (HPF) that the PapaMBlue, produced significantly more *ROS* than free MB. As the radiant exposure increased, the amount of ROS also increased for both groups, as expected, but the (PapaMBlue) produced significantly more (p < 0.0001 and p < 0.0001 for *SOSG* and *HPF*, respectively). The explanation for this somewhat surprising observation is that MB forms dimers and oligomers in aqueous solution that reduces the quantum yield of *ROS* due to quenching of excited states occurring between the individual dye molecules^34^. The addition of the papain protein to the aqueous MB solution presumably binds to the individual MB molecules and thus prevents them to dimerize and forming quenched oligomers. Several studies have previously reported that MB and similar phenothiazinium salts are susceptible to formation of dimers and oligomers and that this inhibits the PDT activity^34^,^35^,^36^,^37^.

PapaMBlue and MB were applied to fibroblasts in the dark in order to evaluate any dark-cytotoxicity *‘in vitro’*. This was the first step to use the new papain-gel with blue dye for PDT. The dermal fibroblast lineage is similar to the lineage of dental pulp fibroblasts and has relevance to the structural tissue organization of dentine. The results are shown in Fig. 4. Using both viability assays it was apparent that the values increased with increasing dye concentration. Using the MTT assay there was a linear increase up to 20 μM MB equivalent, while for the PrestoBlue assay the increase leveled off at 10 μM MB equivalents. The explanation for this increase in measures of viability, we believe can be explained by the tendency of MB to localize in mitochondria where it can act as an acceptor in the electron transport chain^38^,^39^. Since both assays (MTT and PrestoBlue) rely on measurement of mitochondrial activity this increase can be explained by MB causing an increase in electron transport in the mitochondria. There was no statistical difference between the MB and PapaMBlue groups using either viability assay. The MTT assay (Kruskal-Wallis p = 0.8) and the PrestoBlue assay (Kruskal-Wallis p = 0.4).

We used confocal microscopy with organelle-specific green fluorescent probes and blue nuclear stains to examine the sub-cellular localization in cells cultured with MB or PapaMBlue (red fluorescence). Figure 5 shows the three-color overlays (*Blue* = Nuclei, *Green* = Mitochondria, *Red* = MB/PapaMBlue). Figure 5 also shows the three-color overlays (*Blue* = Nuclei, *Green* = Lysosomes, *Red* = MB/PapaMBlue). It is known that the cationic dye MB can localize both in mitochondria and in lysosomes. This is confirmed by our studies and it can be seen that there were no major differences between MB and PapaMBlue. In the PDT images Fig. 5A4,B2,C4,D2 it can be seen that there is less red fluorescence due to photobleaching of the MB dye.

The antimicrobial CFU assays using PDT against *Streptococcus mutans* biofilms showed difference between all groups. There were three dark groups (Fig. 6A). Chlorhexidine 0.12% (CHX) was used as a positive control group because it is the gold standard antibacterial substance used in clinical practice. It was able to reduce the survival fraction by almost 3-logs of CFU, The other dark controls (MB and PapaMBlue without light) gave less than 1-log reduction in CFU of *S*. *mutans*. There were five groups that received light (Fig. 6B). Light alone no PS showed no reduction of CFU. With a fluence of 150 J/cm^2^ only the MB 20 μM group showed a reduction in CFU of two logs. When the fluence used was 200 J/cm^2^, the groups MB 20 μM, PapaMBlue 10 μM and PapaMBlue 20 μM were killed by 2-logs. (p = 0.02, 0.03 and 0.01 respectively, Kruskal-Wallis, post hoc Student-Neuman-Keus) (Fig. 6B).

The images of the biofilms of *S*. *mutans* with (Live dead assay) were visualized by confocal laser scanning microscopy (CLSM) using the live-dead stain (Fig. 7). Propidium iodide (PI) enters more into the membranes of dead cells showing red fluorescence. The green (*Syto9*) dye enters only intact membranes, showing viable cells. The control group showed viable *S*. *mutans* stained with green, (Fig. 7-F1), the (Fig. 7-F2) shows the light alone control, and (Fig. 7-F3) (PapaMBlue, non-light) both displaying a yellow biofilm with some green dots showing some loss of viability. Chlorhexidine 0,12% was used as the antimicrobial gold standard (Fig. 7-F4) and showed a deep red staining indicating complete loss of viability. The PapaMBlue 20 μM activated with two different fluences (150 and 200 J/cm^2^) (Fig. 7-F5,F6) showed a moderately red biofilm indicating a high loss of viability. The live dead staining results were in agreement with the CFU results (Fig. 7), except for the light alone control which showed no loss of CFU but some loss of viability with live dead staining.

Antimicrobial PDT action on biofilms grown on membrane of collagen type I, discussed in the larger context of the evolving relationship with collagen matrix on dentin. It was hypothesized that the PapaMBlue with red light 660-nm resource was used with the purpose to show whether aPDT could kill *S. mutans* biofilms grown upon their natural substrate (type-I collagen). Figure 8 shows the images of *S. mutans* biofilms grown on collagen membranes stained with hematoxylin and eosin (*H&E*). (Fig. 8A1,A2). The chlorhexidine group (Fig. 8-A4) and the PDT groups with MB 20 μM (Fig. 8A3), PapaMBlue 10 μM and PapaMBlue 20 μM (Fig. A5,A6) groups show scanty microorganisms diffusely located within the fibers.

The *S. mutans* images visualized with transmission electron microscopy (*TEM*) revealed intact and smooth cell membrane of the bacteria in dark and light control groups (Fig. 9A,B,E,F). The *S. mutans* cells in the PDT groups with PapaMBlue and MB with different concentrations showed a marked disruption of the cell membrane, with thickened granular aspect. The PapaMBlue 10 and 20 μM treatment groups with 200 J/cm^2^ showed granular intracellular electron-lucid changes with retraction of the intracellular contents accompanied by indentation of the cell envelope. The same effect in a smaller cytosolic area was observed with MB at 10 and 20 μM and 200 J/cm^2^, (Fig. 9G,H). A previous study showed TEM images of *S. mutans* that had been killed by a combination of cold plasma and gold nanoparticles^40^.

The PapaMBlue combined to irradiation demonstrated to be more effective in the removal of the caried tissue in comparison with the control group and the PapaBlue group without irradiation. This fact suggests that PapaMBlue is more effective following irradiation. Papacarie and irradiated Papacarie demonstrated the same effectiveness in the removal of carious tissue (Fig. 10).

The integrity of collagen triple helix is determined from the ratio of absorbance bands (1235 cm−1/1450 cm−1) (Amide III/Pyrrolidine rings). Amide band is sensitive to the presence of secondary structure of collagen, in contrast to Pyrrolidine rings that are independent of collagen structure^41^. Although a slight reduction in the intensity of the amide I, II and III bands on PapaMBlue indicated a direct conformational change in the triple helix of the collagen, the absorbance ratio values >0.8 corroborated the lack of significant denaturation of the collagen (Table 3). Moreover, activation with low-level laser (±660 nm) exerted little effect on this process (Fig. 11), regardless of the energy used for irradiation.

## Conclusion

The present study has shown that the absorption spectra of methylene blue and PapaMBlue are essentially identical. PapaMBlue is equally effective as methylene blue alone in destroying *S. mutans* biofilms without affecting fibroblasts. There was no alteration on the chemical structure of collagen type I. The innovation offered by adding methylene blue to a papain-gel (PapaMBlue) to make a new product is that the PapaMBlue produced significantly more *ROS* than free methylene blue, and this can improve bacterial killing. That shows PapaMBlue, could be a potential for PDT applications in infected dentin.

## Additional Information

**How to cite this article**: Silva Júnior, Z. S. *et al*. Papain gel containing methylene blue for simultaneous caries removal and antimicrobial photoinactivation against *Streptococcus mutans* biofilms. *Sci. Rep.* **6**, 33270; doi: 10.1038/srep33270 (2016).

## Figures and Tables

**Figure 1:**
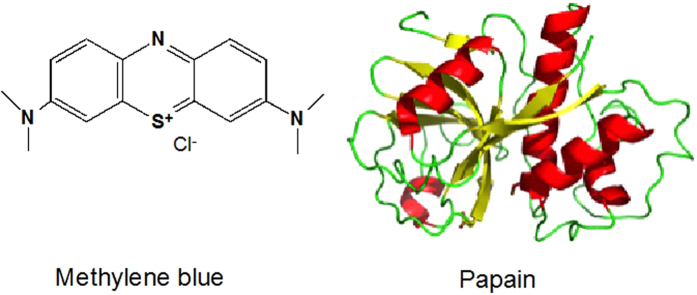
Chemical structures of methylene blue and papain.

**Table 1:** Design of study groups.

Groups	PDT Treatment	
G1 Control negative	No	
G2 (Control Light)	150 J	200 J
G3 (Chlorhexidine 0.12% )	No	
G4 (Methylene Blue 10 μM – Dark)	No	
G5 (Methylene Blue 20 μM-Dark)	No	
G6 (PapaMBlue 10 μM-Dark)	No	
G7 (PapaMBlue 20 μM-Dark)	No	
G8 (Methylene Blue 10 μM-PDT)	150 J	200 J
G9 (Methylene Blue 20 μM-PDT)	150 J	200 J
G10 (PapaMBlue 10 μM-PDT)	150 J	200 J
G11 (PapaMBlue 20 μM-PDT)	150 J	200 J

**Table 2:** Description of the experimental groups used for the second phase of this study.

Group	Treatment	Description
G1_col_	Washing with Milli-Q water (negative control group)	No treatment; samples washed in Milli-Q water for 30 s and dried with absorbing paper
G2_col_	Papacárie	Application time: 5 min; samples washed in Milli-Q water for 30 s and dried with absorbing paper
G3_col_	PapaMBlue	Application time: 5 min; irradiated for 8 min with red laser 20 J/cm^2^. Samples washed in Milli-Q water for 30 s and dried with absorbing paper

**Table 3:** Analysis of the integrity of collagen triple helix.

	Absorbance of1235 cm^−1^	Absorbance of1450 cm^−1^	Absorbance Rate(integrity of triple helix)
Pure collagen	0.88	0.84	1.04
Collagen treated with Papacarie	0.57	0.71	0.81
Collagen treated with PapaMBlue	0.56	0.70	0.73
